# A GIS based approach to neighbourhood physical environment and walking among adults in Colombo municipal council area, Sri Lanka

**DOI:** 10.1186/s12889-021-10983-7

**Published:** 2021-05-26

**Authors:** Shreenika De Silva Weliange, Dulitha Fernando, Shanthi Withanage, Jagath Gunatilake

**Affiliations:** 1 Department of Community Medicine, University of Colombo, Colombo, Sri Lanka; 2 Postgraduate Institute of Science, University of Peradeniya, Peradeniya, Sri Lanka; 3 Department of Geology, University of Peradeniya, Peradeniya, Sri Lanka

**Keywords:** Adults, Colombo, Geographic information systems, Neighbourhood, Physical environment, Walking

## Abstract

**Background:**

Physical Activity (PA) promotes health and wellbeing and walking is one of the easiest and commonest way to incorporate activity into everyday life. This study examined the association between the objectively measured neighbourhood physical environment and walking among the adults in Colombo Municipal Council (CMC) area in Sri Lanka.

**Methods:**

A cross sectional study was carried out and primary data collection carried out to assess walking, socio-demographic characteristics and geo location of residence. Secondary data was obtained to assess neighbourhood environment from existing spatially referenced data from the survey department of Sri Lanka. Geographic Information Systems (GIS) was used to calculate density measures (residential density, land use, connectivity and access) and distance measures, which were then correlated with walking.

**Results:**

A sample consisted of 284 adults aged 29–59 years with a mean age of 40.6 (SD,10.9) years. The total mean minutes walked a week was 175.8 min with a standard deviation of 214.0. The mean and the standard deviations of walking per week for transportation and leisure were 158.4 (SD,196.6) and 17.5 (SD,70.1) respectively. Residential density measures, building foot print area, length of major roads, number of intersections, number of bus stops, and distance to nearest major roads showed significant moderate degree of correlations with total minutes of walked per week, (*p* < 0.001). These results were similar with walking for transportation.

**Conclusion:**

There is indication that neighbourhood features are associated with walking among the adults in the CMC area using secondary data on physical environment. This paves way for further exploration to understand the relationship between neighbourhood physical environment and walking which could be used for effective interventions to promote walking.

## Background

The burden of non-communicable diseases (NCDs) are on the rise in the developing countries and the mortality is reported to be 20–50% higher in Sri Lanka, than in many developed countries [1]. Among the many NCD risks, physical inactivity is the fourth leading risk factor for death in the world [2] and being inactive is known to increase the risk of cardiovascular disease, some cancers, type 2 diabetes, stroke, some mental illnesses and premature death [3–5]. In Sri Lanka, the prevalence for physical inactivity was observed to be 22.5% for males and 38.4% for females from the Non-Communicable Disease Risk Factor Survey in 2015 [6] while in district of Colombo the prevalence of inactivity was 18.0% for males and 20.3% for females [7].

Out of the many forms of physical activity, walking is one of the most common forms of activity carried out by adults. Many factors are known to influence walking, including personal and environmental factors. In describing the factors in the built environment for PA and walking, measures such as residential density, land use, intersection density, street connectivity and access and distance to facility is considered important [8]. Commonly used concepts in literature are “proximity”, “connectivity” and “urban design” [9]. Proximity refers to how close different travel destinations are to one another in space and is operationalized in terms of “density” and “land use mix”. Density measures the concentration of people, dwelling units or households, while land use mix refers to the spatial placement of different types of land uses (industrial, residential, commercial). When the land uses are mixed, there is greater number of destinations that are close to a person’s home or office. “Connectivity” refers to the number and directness of transportation linkages between destinations [10]. A highly connected neighbourhood has many linkages between destinations and is usually defined in terms of number of road intersections in an area. A highly connected neighbourhood provides more route options for travellers and shortens trip distance, thereby influencing people to use non-motorized forms of transportation. Many environment attributes such as street connectivity, good access to destination such as to recreational facility and public transport have shown relationship with walking in the previous studies [11–17].

To assess this relationship the physical environment must be measured ether by observations of the environment through audits, perception of the environment by individuals or by using secondary data on environment. Recently with the advancement of digital maps and the Geographic Information Systems (GIS) technology, objective measures to assess the environment have been widely used in the area of PA, giving the capability to assess spatial relationships which could be useful to solve public health issues [18]. An added advantage of GIS based analysis is that that publicly available geospatial data could be a more feasible and cost-effective method for exploring relationships [19].

When using GIS to assess relationship with walking, density and distance measures should be ideally measured through network analysis. However, in situations where available data are not compatible for network analysis due to incompleteness of the network structure the recommendation is for straight line analysis [20]. The twin cities walking study environment and physical activity: GIs protocol, gives in details the common attributes that could be operationalised, also giving the concept, formulae, approach, and steps in calculating these attributes using geo referenced data [20]. Some common environmental attributes that could be assessed using GIS are population per unit area, street lights and street trees within a unit area, distance to facility, sidewalk length, building foot print area, intersections and road length per unit area etc. A GIS Based assessment of physical environment across 12 countries have been carried out assessing residential density, street connectivity, mix of land uses, and access to public transit, parks, and private recreation facilities [21] and its relationship with PA. As Sri Lanka experiences rapid urbanisation leading to changes in the built environment, it is vital that assessment of built environment and its association with walking be explored as the country progresses to achieve the targets of sustainable development goals for 2030 agenda by reducing one third of premature mortality from NCD [22]. The aim of this study was to explore the relationship between neighbourhood environment and walking among the adults in Colombo Municipal Council (CMC) area in Sri Lanka.

## Methods

### Study aim, design and setting

This study to explore the relationship of walking with GIS based neighbourhood environment was a subcomponent of a cross sectional study conducted in the district of Colombo with a sample of 1320 to assess the prevalence and correlates of PA, reporting that 79.4% walked for transport and only a mere 14.5% walked for leisure [7]. The GIS analysis was carried out only in the CMC area, within the district, which is the island’s economic capital, by far the country’s biggest city, and the most urbanised. The aim was to report on the objectively measured physical environmental factors associated with walking using GIS. The main reason for selecting only the CMC area for GIS analysis was the availability of digital data such as the road structure, land use and other services making it possible to carry out environmental data analysis using GIS. Data collection was carried out from September 2010–February 2011.

### Selection of participants

**Fig. 1:**
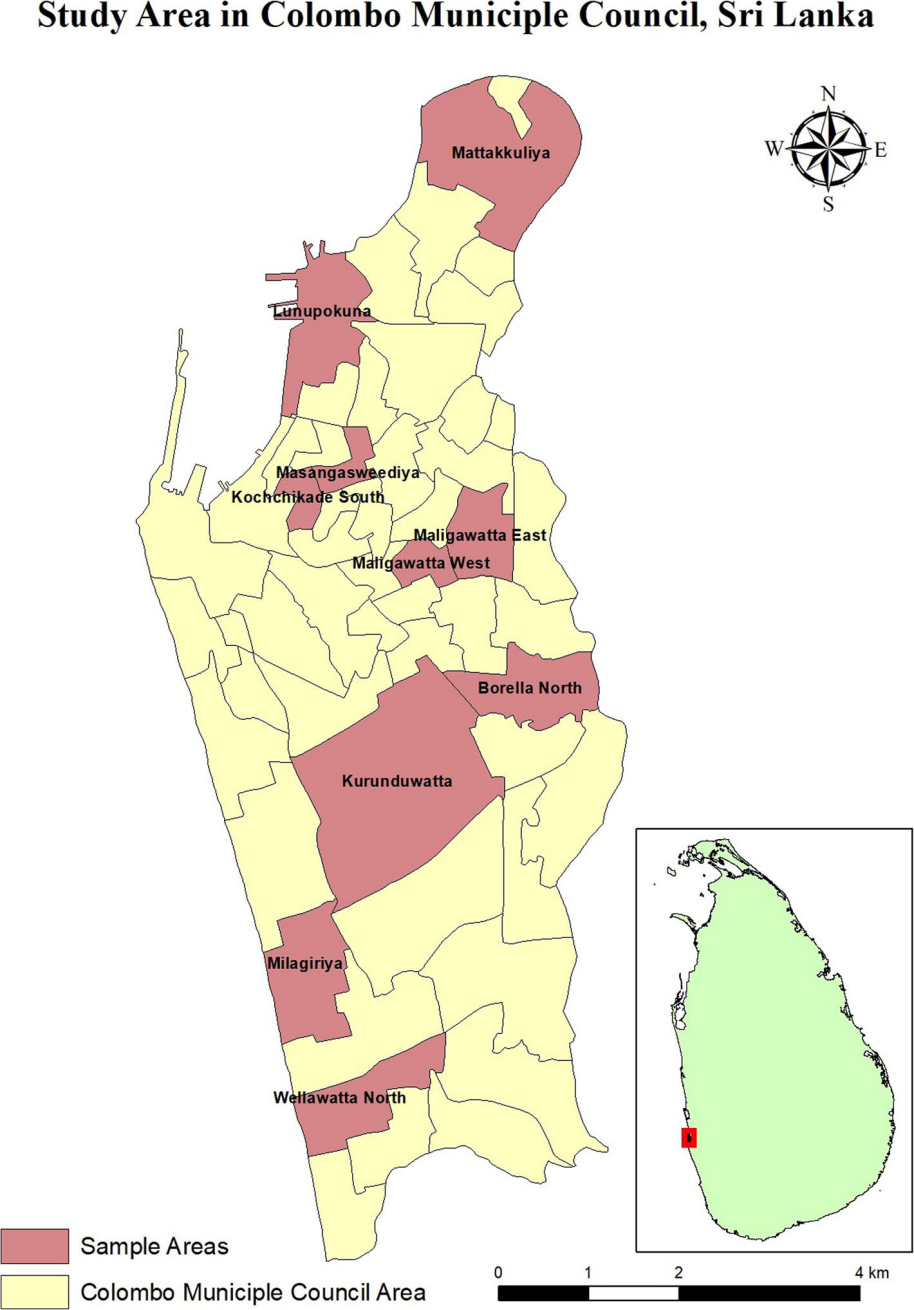
CMC Neighbourhood Study map

The sample was selected using a cluster sampling method with the primary sampling unit being a Grama Niladhari Division (GND), the smallest administrative entity for which information on socio demographics are produced by Department of Census and Statistics in Sri Lanka. The GNDs selected are shown in Fig. 1. From each GND 40 households were selected. The detailed sampling mechanism has been described previously (7).

### Primary data collection

#### Survey measures

The study instrument was a reliable and pre-tested interviewer administered questionnaire. Socio-demographics measures that were assessed were the age, sex, ethnicity, civil status, educational level, average monthly household income and self-rated health status. Walking was assessed using the validated International Physical Activity Questionnaire, Long Form (IPAQ) [23]. It is a widely used, standardized instrument to measure the habitual practice of PA of populations over the last 7 days [23–25]. Participants reported the frequency and usual duration of each type of activity including walking, undertaken during the previous week in the different domains of job, transport, domestic/garden chores and leisure. However, for this analysis only the transport and leisure related walking was selected as the neighbourhood environment features are mostly related to them [17, 21].

#### Location measures

The data collectors with a medical background were trained on measuring the coordinates of the housing location. After completing the questionnaire, the coordinates of the locations were recorded using Megallon eXplorist 510 GPS units.

### Secondary data collection

Population estimates from the most recent census data in Sri Lanka was used to determine population density [26]. Neighborhood environment characteristics were obtained from the Colombo land use maps (1:50,000) which were the recent most data which were, geocoded and verified by the Survey Department, Sri Lanka. The details of the digital data layers including the scale and format are shown in Table 1. No parcel data were available.

**Table 1:** Description of the digital data from the Survey Department 2010

Layer	Scale	Format
10 m Interval Contours	10 m	DGN and geo database
Spot Heights	11111 m	DGN and geo database
Streams and Rivers	1: 10,000	Coverage
Land Use	1: 50,000	Coverage
Roads	1: 50,000	Coverage
District, DSD, and GND	1: 250,000 and 1: 50,000	Shape files and Coverage
Buildings	1: 10,000	DGN format

Using the above secondary data sources the following density and distance measures were calculated.

#### Density measures

Density measures were carried out within the 200 m, 400 m and 600 m buffer limit of the participant’s residence. All buffers were straight-line buffers as the network structure was incomplete for Colombo. The neighbourhoods were defined by creating a 600 m radius “straight line” around each geo-coded participant’s address. Small radii of 200 m and 400 m were also evaluated as it was hypothesised that a smaller area around one’s home might be more influential in individual’s choice to walk [18]. Population and housing density was calculated using data from the national census (26). The built area, access and connectivity density measures were calculated from the digital data from the survey department. The land use measure is a measure of ground covered with buildings, which cannot be used for walking. The number of buildings and the building foot area were then calculated within the buffer zones. Road lengths (both main and other roads) per unit area, intersections per unit area, and number of bus stops per unit area were used to assess access and connectivity.

#### Distance measures

Distance to the nearest major roads, nearest beach and the nearest park was calculated using the data from the survey department (Table 1) which were all were straight-line distances as network work structures were not available.

### Analysis

#### GIS spatial analysis

Quality of data was verified before GIS spatial analysis. The secondary data that were collected were the most recent data. However, these were not assessed for count error (incomplete data), attribute error (inaccurate classification of facilitates or characteristics) and the positional error (inaccurate geo coding) due to logistic constraints.

The primary data collected, were rechecked for its accuracy in 10 housing locations by the experts in GPS. The coordinates of the household were downloaded from the GPS units using the vantage point software and was further visualised using the OZI explorer software. Thereafter, it was converted to shape file format as a point layer by the OZI explorer software. These were converted from their original spatial reference parameters of WGS84 to Kandawala system to be appropriate for Sri Lanka.

Initially, all the spatial data were converted in to ArcGIS geodatabase data model. Thereafter, a feature dataset was created. ArcGIS 9.3 software and its extensions such as 3D Analyst, Network Analyst, and Data interoperability were used in the study. Further, free extensions available such as Hawth’s tools, X tools etc. were also used for the various spatial analysis in the study. The physical environmental variables were created and were based on the literature available, especially the GIS protocol developed for the Twin City study [20]. The concept and formulae in conducting GIS spatial analysis, for the indices are outline in Table 2.

**Table 2:** Concept and formulae for the neighbourhood environment attributes

Indices	Concept	Formulae
Density measures for the total buffer area	Population density	Number of persons within the buffer / Total buffer area
	Housing density	Number of housing units within the buffer / Total buffer area
Density measures for the residential area	Population density	Number of persons within the buffer / Total residential buffer area
	Housing density	Number of housing units within the buffer / Total residential buffer area
Built area measures	Number of buildings per total buffer area	Total number of buildings per buffer area
	Building foot print area within the total buffer area	Building footprint area in the buffer excluding vacant or agricultural land uses.
Access and connectivity	Length of major roads within the total buffer area	Length of major road with both interstates and ramps removed per total buffer area
	Length of other roads within the total buffer area	Length of other road with both interstates and ramps removed per total buffer area
	Number of intersections within the total buffer area	Number of intersections within the buffer area
	Number of bus stops within the total buffer area	Number of bus stops within the buffer area
Distance to facility	To nearest major roads	Straight line distance to nearest major roads
	To nearest beach	Straight line distance to nearest beach
	To nearest park	Straight line distance to nearest park

#### Analysis of walking

Total number of minutes of walking carried out during transportation and leisure was calculated as per the IPAQ protocol (23, 24).

### Statistical analysis

Statistical analysis was performed using SPSS version 17. The physical environmental variables created through the GIS systems for each individual was correlated with his/her minutes of walking a week. Spearman correlation coefficient was calculated as the data were not normally distributed. A threshold of 0.05 for statistical significance was used.

## Results

### Sample characteristics

Table 3 describes the socio-demographic characteristics of the study sample. In total, 284 (71%) participants with mean age of 40.6 years (SD = 10.9) provided complete data after excluding participants with missing data. The sex ratio of male to females was 1: 1.3. More than half the sample had an education of above G.C.E O/L, with less than 1% having no formal education.

**Table 3:** Socio-demographic and perceived health of the study sample (*n* = 284)

Socio- Demographic characteristic	No.	%
**Age (years)**		
20–29	49	17.3
30–39	84	29.6
40–49	77	27.1
50–59	74	26.0
**Sex**		
Male	126	44
Female	158	56
**Education (highest achievement)**		
No formal education	1	0.4
Grade 5	30	10.6
Grade 6-grade10	105	37.0
General Certificate of Education Ordinary level (G.C.E.O/L)	100	35.2
General Certificate of Education Advanced Level (G.C.E.A/L)	33	11.6
Higher education (University/diploma/technical)	15	5.2
**Average monthly household income (in Sri Lanka Rupees)**		
30,000 or less	233	82.0
More than 30,000	51	18.0
**Self-rated health status**		
Excellent	7	2.5
Very good	44	15.5
Good	111	39.1
Fair	116	40.8
Poor	6	2.1

### Pattern of walking

The pattern of walking among the study sample is shown in Table 4. The median minutes of walking for transport were 80 min a week (IQR 10–210). The median, and the interquartile range for the minutes walked a week for leisure was zero as only 10.5% of the persons walked for leisure. The median minutes of total walking among males were more than the females and more in the high-income category. Males walked more than females in total. Walking for leisure was observed more among the higher income group and among males. Walking for transport was observed more in the lower income category.

**Table 4:** Distributions of the pattern of walking by selected socio demographic factors

Characteristics		Walking for Leisure Minutes per Week (IQR)		Walking for Transport Minutes per Week (IQR)		Total walking- Minutes per Week (IQR)	
		Mean (SD)	Median (IQR)	Mean (SD)	Median (IQR)	Mean (SD)	Median (IQR)
**Sex**	Male (*n* = 126)	23.2 (74.8)	0 (0–0)	158.9 (189.6)	90 (19–210)	182.1 (210.6)	140 (20–280)
	Female (*n* = 158)	13.0 (66.2)	0 (0–0)	157.9 (202.6)	78 (0–210)	170.9 (217.2)	90 (0–218)
**Age Group**	18–29 (*n* = 49)	12.8 (66.5)	0 (0–0)	161.7 (179.1)	100 (0240)	174.6 (184.6)	140 (0–130)
	30–39 (*n* = 84)	9.0 (46.3)	0 (0–0)	122.8 (178.2)	60 (0–175)	131.9 (185.9)	60 (0–173)
	40–49 (*n* = 77)	24.9 (80.9)	0 (0–0)	171.7 (215.5)	70 (20–225)	196.6 (236.8)	120 (30–280)
	50–59 (*n* = 74)	22.5 (81.9)	0 (0–0)	182.6 (205.2)	120 (18–300)	205.1 (232.1)	130 (20–330)
**Income**	Rupees 30,000 or less (*N* = 233)	10.9 (60.8)	0 (0–0)	161.1 (201.5)	80 (10–210)	172.0 (221.1)	80 (10–230)
	More than 30,000 Rupees (*n* = 51)	47.6 (97.7)	0 (0–0)	145.9 (173.6)	90 (0–210)	193.5 (178.8)	150 (30–315)
**Total**	N = 284	17.5 (70.1)	0 (0–0)	158.4 (196.6)	80 (10–210)	175.8 (214.0)	105 (20–270)

### Distribution of the neighbourhood environment attributes

The physical environment attributes were assessed around 200 m, 400 m and 600 m buffers and all showed a non- normal distribution. The values for the 200 m buffer are shown in Table 5, as the 200 m is the most likely walked area within the neighbourhood. The mean housing densities within the residential areas was considerably higher compared to total areas depicting that there might be a large areas where non-residential buildings are present. The mean length of minor roads were more than 4 times that of the major roads showing that adequate street structure is available. It was also seen that the mean number intersections and bus stops were 12.3 and 18.5 respectively within a 200 m buffer area also showing that access was high in the CMC area.

**Table 5:** Descriptive statistics of physical environment attributes within 200 m buffer area (n = 284)

Physical environment attributes	Unit	Mean	Standard error of mean	Standard Deviation	Skewness	Kurtosis
**Density measures within a 200 m buffer area**						
Housing density	Houses/km^2^	3647.8	119.9	2021.2	0.5	−0.8
Population density	Persons/km^2^	20,286.2	728.0	12,269.08	0.5	−0.9
**Density measures per residential area within a 200 m buffer area**						
Housing density	Houses/km^2^	203,563.8	175,692.3	255,602.3	16.8	282.2
Population density	Persons/km^2^	989,235.0	844,940.0	142,141.7	16.8	282.2
**Land use measures**						
Number of buildings within the total buffer area	Units	141.6	4.6	78.1	1.8	2.1
Building foot print area within the total buffer area	Units	0.1	0.0	0.0	1.3	2.1
**Access and connectivity**						
Length of major roads within the total buffer area	m	544.9	26.0	439.6	0.2	−1.1
Length of minor roads within the total buffer area	m	2607.2	36.1	608.6	−0.1	−1.3
Number of intersections within the total buffer area	Units	12.3	0.5	7.9	0.7	−0.3
Number of bus stops within the total buffer area	Units	18.5	0.6	10.4	0.9	0.11

### Association between neighbourhood physical environment and walking

Table 6 shows that all residential density measures, number of intersections, number of bus stops, and distance to nearest major roads and beach showed positive moderate correlations with total walking, in the 200 m buffer zone which was statistically significant. The results were almost similar with walking for transportation with a slightly higher correlation coefficient. A negative significant correlation was observed with the building foot print area, length of major roads for the total walking. Similar correlations were observed in the 400 m and 600 m buffers. However, most environment features did not show moderate significant correlations with walking for leisure.

**Table 6:** Association between neighbourhood physical environment and walking

	Physical environmental attributes	Transportation walking minutes a week		Leisure time walking minutes a week		Total walking minutes a week	
		r	***p*** value	r	P value	r	p value
**Density measures (at 200 m buffer)**							
**Residential Density**	Housing density per total buffer area	0.33	0.001**	− 0.40	0.499	0.29	0.001**
	Population density per total buffer area	0.33	0.001**	−0.03	0.568	0.29	0.001 **
	Housing density per residential area within buffer	0.40	0.001**	−0.16	**0.01** **	0.34	0.001**
	Population density per residential area within buffer	0.39	0.001**	−0.17	0.003*	0.33	0.001**
**Land use**	Number of buildings within the total buffer area	−0.09	0.12	0.17	0.004*	−0.06	0.35
	Building foot print area within the total buffer area	0.21	0.001**	−0.14	**0.02** *	−0.23	0.001**
**Access / connectivity**	Length of major roads within the total buffer area	−0.43	0.001**	−0.10	- 0.09	−0.43	0.001**
	Length of minor roads within the total buffer area	−0.03	0.58	−0.01	0.98	−0.02	0.75
	Number of intersections within the total buffer area	0.29	0.001**	−0.15	**0.02** *	0.25	0.001**
	Number of bus stops within the total buffer area	0.26	0.001**	−0.08	−.16	0.23	0.001**
**Distance measures – straight line**							
Distance to nearest major road		0.20	0.001**	0.19	0.03	0.20	0.001**
Distance to nearest beach		0.14	**0.02** *	0.19	**0.01** *	0.18	0.001**
Distance to nearest grounds /park		0.02	0.77	−0.15	**0.02** *	−0.02	0.74

r: Spearman r

p: probability

**- significant at p < 0.001

*- significant at *p* < 0.05

## Discussion

Few studies have examined the relationship of the built environment and walking in developing countries through a GIS approach. This study is one of the first to examine this association in Sri Lanka. A cross sectional study methodology was adopted like many other studies looking at walking and its association with objectively measured physical environmental attributes using GIS [18]. This study adopted the GIS concepts and formulae derived from the GIS protocol developed for the Twin City Study [20] and adjusted it according to the availability of secondary data. Although, the environment data were not collected for the purpose of studies related to PA, similar approaches have been used in many other studies where the secondary data were gathered for other purposes [27, 28] were used for analysis as collecting environment related spatially referenced data is expensive.

It is seen from the current study that most of the walking carried out by the participants were related to transport. Only a very minimal amount of walking was carried out for leisure, and that only less than 25% walked for leisure indicated by the interquartile ranges for leisure time walking. This might be due to the fact that in Sri Lanka people do not engange routinely in physically active leisure be it walking or other forms of activity [7]. Further it is seen that males and higher income group had a higher median in total minutes of walking carried out in a week. However, when distribution of the transport related walking was considered the mean minutes of walking per week was higher in the low-income category. This might be due to the fact that lower income category commonly uses public transportation and walking to meet their transport requirement, and the higher income group might be walking for leisure which is evident from the mean scores. Similar observations are there in other studies where people in low-income households were twice as likely to walk compared to the higher income households [27]. Similarly, in China it was seen that level of total PA was higher among the low socio-economic group [29]. Both male and female respondents were less likely to engage in walking for leisure than walking for transport. However, males engaged more in walking for leisure than females. This may be due to the different roles that the males and females play in the household or due to the fact that the environment being more conducive for the male gender especially in terms of safety or social acceptance for physical activity which was seen in a qualitative inquiry carried out in Colombo, Sri Lanka [30]. The neighbourhood environment features that were operationalised for this study showed a wide variation when assessed for each as shown by the wide standard deviation, indicating the possibility that the selected neighbourhoods varied much in terms of theses environment variables. Further, the variables that were selected and operationalised were in alignment with perceived environment variables that that was selected for the scale developed and validated to assess the perceived physical and social environment associated with physical activity for Sri Lankan Adults [31].

In the present study, mild to moderate positive correlations were observed between minutes of walking for transport and total walking with residential density and connectivity at the 200 m buffer. This was similar to that observed in the Twin city study where the correlation coefficients ranged between 0.3–0.5 [27]. This could be due to people engaging in more walking when there are diverse destinations and the neighbourhood is more connected. Similarly, a study done in Atlanta in 2003 [9] which assessed the net residential density (number of residential units per residential area), street connectivity (number of intersections per unit area) and land use mix (evenness of distribution of square footage of residential and commercial development) through a GIS database, showed that all of the above indicators were positively associated with the number of minutes of moderate PA per day.

The present study has limitations that should be recognized. As this study was a cross-sectional design, it prevents assessment of causality. The association of environment for leisure time walking was less concusive, possibly due to the fact that there were only few participants who engaged in waliking for leisure. Therefore, the built environment for leisure related walking needs to be further explored adopting a different methodology to compare perception of environment among those walking for leisure and those not walking for leisure. Yet, the current study generated interesting relationships between the built environment and transportation related walking. However, it is important that the findings be viewed in the backdrop of a small sample size which could have driven the possible relationships. The GIS based study had only five environmental factors and was not able to capture some important environmental variables that often are not available in GIS databases such as quality of the sidewalks, safety in the neighbourhood environment, presence of shade, traffic safe etc. which are known to be associated with walking [13–16]. However, it gives the capability to explore the walking and the environment in relation to walking using already available secondary data. The use of secondary data involved certain limitation to the analysis. Network analysis could not be carried out due to incomplete networks in the secondary data that were available. Yet, collecting primary data and carrying out objective measurement through geo-coding was not feasible due to logistic constraints in the present study as would be in many explorative studies. Therefore, this GIS study explored the possibility of using GIS based data for future studies related to PA and the physical environment in developing countries with limited resources.

This study carried many strengths, of which one is the use of a valid measurement of walking. This was assessed through a widely accepted tool, the IPAQ long form, which was also validated for the local setting previously [23] giving good validity and reliability measures. Another was making use of publicly available data for health-related research. Thirdly, this study used a similar approach is assessing the association between PA and the GIS based physical environment attributes such as residential density, land use, connectivity and used a correlation analysis [18, 27] enabling comparisons [28]. Further, the use of three network buffers specific to neighbourhood is a key strength of this analysis. Although the 200 m buffer is presented here all three buffer-specific analysis gave near comparable results. Neighbourhoods were defined around a radius of 200 m, 400 m and 600 m from the participants residents, which was also the method adopted in the Twin City study [20].

## Conclusions

This was the first Sri Lankan study to examine the walking and objectively measured environment attributes in adults. There is an indication that neighbourhood features are associated with walking in Sri Lankan adults. It can be concluded that GIS based studies could be adopted in Sri Lanka in understanding how neighbourhood physical environment attributes for walking are associated with the walking pattern. This understanding could help in designing effective interventions to promote walking and thereby make the people more active and healthier.

## Data Availability

The datasets used and/or analysed during the current study are available from the corresponding author on reasonable request.

## Abbreviations

CMC: Colombo Municipal Council
G.C.E.A/L: General Certificate of Education Advanced Level
G.C.E.O/L: General Certificate of Education Ordinary level
GIS: Geographic Information Systems
GNDs: *Grama Niladhari* Divisions
GPS: Global Positioning System
IPAQ: International Physical Activity Questionnaire
IQR: Interquartile Range
NCDs: Non-Communicable Diseases
PA: Physical Activity
